# The effect of frozen embryo transfer regimen on the association between serum progesterone and live birth: a multicentre prospective cohort study (ProFET)

**DOI:** 10.1093/hropen/hoac054

**Published:** 2022-11-28

**Authors:** Pedro Melo, Simon Wood, Georgios Petsas, Yealin Chung, Christina Easter, Malcolm J Price, Simon Fishel, Mohammed Khairy, Charles Kingsland, Philip Lowe, Madhurima Rajkhowa, Victoria Sephton, Shilpi Pandey, Rahnuma Kazem, David Walker, Julija Gorodeckaja, Mark Wilcox, Ioannis Gallos, Amanda Tozer, Arri Coomarasamy

**Affiliations:** Tommy’s National Centre for Miscarriage Research, Institute of Metabolism and Systems Research, College of Medical and Dental Sciences, University of Birmingham, Edgbaston, UK; CARE Fertility Birmingham, Edgbaston, UK; CARE Fertility Chester, Chester, UK; CARE Fertility Sheffield, Sheffield, UK; Tommy’s National Centre for Miscarriage Research, Institute of Metabolism and Systems Research, College of Medical and Dental Sciences, University of Birmingham, Edgbaston, UK; CARE Fertility Birmingham, Edgbaston, UK; Institute of Applied Health Research, University of Birmingham, Edgbaston, UK; Institute of Applied Health Research, University of Birmingham, Edgbaston, UK; NIHR Birmingham Biomedical Research Centre, University Hospitals Birmingham NHS Foundation Trust and University of Birmingham, Birmingham, UK; CARE Fertility Nottingham, Nottingham, UK; Liverpool John Moores University, School of Pharmacy and Biomolecular Sciences, Liverpool, UK; CARE Fertility Birmingham, Edgbaston, UK; CARE Fertility Liverpool, Liverpool, UK; CARE Fertility Manchester, Manchester, UK; CARE Fertility Birmingham, Edgbaston, UK; CARE Fertility Chester, Chester, UK; CARE Fertility Nottingham, Nottingham, UK; CARE Fertility Northampton, Northampton, UK; CARE Fertility Bath, Bath, UK; CARE Fertility London, London, UK; CARE Fertility Nottingham, Nottingham, UK; Tommy’s National Centre for Miscarriage Research, Institute of Metabolism and Systems Research, College of Medical and Dental Sciences, University of Birmingham, Edgbaston, UK; Aria Fertility, London, UK; Tommy’s National Centre for Miscarriage Research, Institute of Metabolism and Systems Research, College of Medical and Dental Sciences, University of Birmingham, Edgbaston, UK; CARE Fertility Birmingham, Edgbaston, UK

**Keywords:** progesterone, frozen embryo transfer, luteal phase support, live birth, miscarriage

## Abstract

**STUDY QUESTION:**

What is the association between serum progesterone levels on the day of frozen embryo transfer (FET) and the probability of live birth in women undergoing different FET regimens?

**SUMMARY ANSWER:**

Overall, serum progesterone levels <7.8 ng/ml were associated with reduced odds of live birth, although the association between serum progesterone levels and the probability of live birth appeared to vary according to the route of progesterone administration.

**WHAT IS KNOWN ALREADY:**

Progesterone is essential for pregnancy success. A recent systematic review showed that in FET cycles using vaginal progesterone for endometrial preparation, lower serum progesterone levels (<10 ng/ml) were associated with a reduction in live birth rates and higher chance of miscarriage. However, there was uncertainty about the association between serum progesterone levels and treatment outcomes in natural cycle FET (NC-FET) and HRT-FET using non-vaginal routes of progesterone administration.

**STUDY DESIGN, SIZE, DURATION:**

This was a multicentre (n = 8) prospective cohort study conducted in the UK between January 2020 and February 2021.

**PARTICIPANTS/MATERIALS, SETTING, METHODS:**

We included women having NC-FET or HRT-FET treatment with progesterone administration by any available route. Women underwent venepuncture on the day of embryo transfer. Participants and clinical personnel were blinded to the serum progesterone levels. We conducted unadjusted and multivariable logistic regression analyses to investigate the association between serum progesterone levels on the day of FET and treatment outcomes according to the type of cycle and route of exogenous progesterone administration. Our primary outcome was the live birth rate per participant.

**MAIN RESULTS AND THE ROLE OF CHANCE:**

We studied a total of 402 women. The mean (SD) serum progesterone level was 14.9 (7.5) ng/ml. Overall, the mean adjusted probability of live birth increased non-linearly from 37.6% (95% CI 26.3–48.9%) to 45.5% (95% CI 32.1–58.9%) as serum progesterone rose between the 10th (7.8 ng/ml) and 90th (24.0 ng/ml) centiles. In comparison to participants whose serum progesterone level was ≥7.8 ng/ml, those with lower progesterone (<7.8 ng/ml, 10th centile) experienced fewer live births (28.2% versus 40.0%, adjusted odds ratio [aOR] 0.41, 95% CI 0.18–0.91, *P *=* *0.028), lower odds of clinical pregnancy (30.8% versus 45.1%, aOR 0.36, 95% CI 0.16–0.79, *P *=* *0.011) and a trend towards increased odds of miscarriage (42.1% versus 28.7%, aOR 2.58, 95% CI 0.88–7.62, *P *=* *0.086). In women receiving vaginal progesterone, the mean adjusted probability of live birth increased as serum progesterone levels rose, whereas women having exclusively subcutaneous progesterone experienced a reduction in the mean probability of live birth as progesterone levels rose beyond 16.3 ng/ml. The combination of vaginal and subcutaneous routes appeared to exert little impact upon the mean probability of live birth in relation to serum progesterone levels.

**LIMITATIONS, REASONS FOR CAUTION:**

The final sample size was smaller than originally planned, although our study was adequately powered to confidently identify a difference in live birth between optimal and inadequate progesterone levels. Furthermore, our cohort did not include women receiving oral or rectal progestogens.

**WIDER IMPLICATIONS OF THE FINDINGS:**

Our results corroborate existing evidence suggesting that lower serum progesterone levels hinder FET success. However, the relationship between serum progesterone and the probability of live birth appears to be non-linear in women receiving exclusively subcutaneous progesterone, suggesting that in this subgroup of women, high serum progesterone may also be detrimental to treatment success.

**STUDY FUNDING/COMPETING INTERESTS:**

This work was supported by CARE Fertility and a doctoral research fellowship (awarded to P.M.) by the Tommy’s Charity and the University of Birmingham. M.J.P. is supported by the NIHR Birmingham Biomedical Research Centre. S.F. is a minor shareholder of CARE Fertility but has no financial or other interest with progesterone testing or manufacturing companies. P.L. reports personal fees from Pharmasure, outside the submitted work. G.P. reports personal fees from Besins Healthcare, outside the submitted work. M.W. reports personal fees from Ferring Pharmaceuticals, outside the submitted work. The remaining authors have no conflict of interest to declare.

**TRIAL REGISTRATION NUMBER:**

ClinicalTrials.gov: NCT04170517.

WHAT DOES THIS MEAN FOR PATIENTS?

Progesterone is a hormone produced by the corpus luteum in the ovary, after an oocyte has been released. If the oocyte is successfully fertilised by sperm, in the fallopian tube, it becomes an embryo. The actions of progesterone enable the uterus to become receptive to the embryo as it implants in the uterine lining. Without progesterone, pregnancy loss is inevitable. There has been research investigating whether blood levels of progesterone are associated with the chance of success in women having treatment with frozen embryos. Most of the existing studies are flawed because they are retrospective, relying on historical data, which may skew study findings. In addition, the available studies have focused mainly on women receiving vaginal progesterone to support the lining of the uterus once a thawed embryo has been transferred.

We performed a prospective study in eight fertility clinics in the UK. Rather than relying on historical data, we approached patients as they presented for treatment and gathered data from those who agreed to take part in our study. We collected blood from participants on the day of frozen embryo transfer and tested the progesterone level to investigate its association with treatment outcomes. We found that low progesterone blood levels (<7.8 ng/ml) were associated with reduced probability of achieving a live birth. However, the association between progesterone blood levels and the probability of achieving a live birth varied depending on the route of progesterone administration. In women receiving vaginal progesterone, higher progesterone levels were associated with a gradual increase in the average probability of a live birth, whereas in women receiving only injectable progesterone, higher progesterone levels may have reduced the chance of a live birth. In participants receiving a combination of both routes (vaginal and subcutaneous injection), the probability of live birth appeared to remain stable as serum progesterone levels increased.

## Introduction

The use of frozen embryo transfer (FET) treatment has become increasingly common around the world in the past decade (Kushnir *et al.*, 2017; Centers for Disease Control and Prevention, Society for Assisted Reproductive Technology, 2018; HFEA, 2020). This stems from evidence showing that the effectiveness of FET is non-inferior to that of fresh embryo transfer treatment (Shi *et al.*, 2018; Stormlund *et al.*, 2020). Furthermore, FET significantly reduces the risk of late-onset ovarian hyperstimulation syndrome (OHSS), which affects up to one in three women undergoing fresh embryo transfer immediately after ovarian stimulation (Mourad *et al.*, 2017). Embryo cryopreservation also allows for the use of add-on therapies such as preimplantation genetic testing for aneuploidy (PGT-A) and the adjustment of transfer timing based on transcriptomic analysis of the endometrium (Simón *et al.*, 2020). These reasons have led some fertility clinics to adopt a universal ‘freeze all’ policy, facilitated by recent advances in cryopreservation and embryo culture techniques (Blockeel *et al.*, 2019).

There are different ways to prepare the endometrium for FET treatment. Embryo thawing and transfer may be undertaken in the luteal phase of a woman’s natural menstrual cycle (NC-FET), with or without progesterone supplementation, or following the sequential administration of HRT with exogenous oestrogen and progesterone (HRT-FET) to mimic a natural uterine cycle (Labarta and Rodríguez, 2020). In FET cycles using progesterone supplementation, this can be administered via different routes, including vaginal, intramuscular, subcutaneous, rectal or oral routes, with varying pharmacokinetics. There remains debate on the route and dosage of progesterone associated with optimum treatment outcomes in FET, with recent studies yielding conflicting results (Shapiro *et al.*, 2014; Devine *et al.*, 2021; Santos-Ribeiro *et al.*, 2021).

Regardless of whether progesterone is synthesised endogenously or exogenously administered, it is widely accepted that in its absence, pregnancy invariably fails (Csapo *et al.*, 1974). This has led some investigators to postulate that the level of serum progesterone around the time of FET may be associated with treatment success (Kofinas *et al.*, 2015; Yovich *et al.*, 2015; Labarta *et al.*, 2017). There is no consensus on this matter, however. Some argue that the uterine levels of progesterone are more critical to pregnancy success than serum measurements, while other researchers advocate that the systemic effects of progesterone are just as important to ensure the right immune-endocrine milieu is present at the time of implantation and placentation (Shah *et al.*, 2019; Labarta *et al.*, 2021b).

There have been several studies investigating the association between serum luteal progesterone and FET outcomes. In a recent systematic review and meta-analysis, we found evidence that low serum progesterone (<10 ng/ml) around the time of FET was associated with reduced odds of ongoing pregnancy or live birth, lower clinical pregnancy rates and higher risk of miscarriage (Melo *et al.*, 2021). However, our primary analyses were restricted to participants having HRT-FET with vaginal progesterone only, due to a lack of data on women undergoing NC-FET and HRT-FET with non-vaginal routes of progesterone administration. In addition, there was uncertainty about whether the odds of treatment success increased linearly as progesterone levels rose. We identified a need for prospective data analysing FET outcomes according to serum progesterone levels in a continuous fashion to account for a non-linear association rather than focusing exclusively on the ‘high’ versus ‘low’ serum progesterone dichotomy. In addition, there remains a paucity of studies investigating the association between serum progesterone and treatment outcomes in women having NC-FET and non-vaginal routes of progesterone for luteal phase support.

Here, we present the results of the first multicentre prospective cohort study aimed at investigating the association between serum progesterone levels and the probability of live birth in women undergoing different FET regimens.

## Materials and methods

### Design

This was a multicentre, prospective cohort study conducted in eight CARE fertility clinics in the UK (Bath, Birmingham, Chester, London, Manchester, Northampton, Nottingham and Sheffield) between January 2020 and February 2021.

### Ethics approval

The ProFET study protocol was approved by the CARE Fertility institutional review board (IRB) committee on 3 November 2019. The IRB panel members were all independent of CARE Fertility and included a university fellow, a senior clinical trials fellow and a senior research nurse. The study protocol was also approved by the medical directors of all participating clinics. We registered the study on ClinicalTrials.gov prospectively on 20 November 2019 (NCT04170517).

We obtained verbal and written consent from all participating women and kept an anonymised study log with reasons for refusal to participate. Women were able to withdraw from the study at any point.

### Study population

We approached all women undergoing FET with blastocyst embryos in the participating sites. CARE Fertility policy restricts fertility treatment to women with body mass index (BMI) <35 kg/m^2^. We excluded women whose clinician had planned serum progesterone testing during FET treatment (without blinding) and those undergoing endometrial receptivity array testing. We analysed only the first FET cycle conducted in each participant for the duration of the study, to allow for analyses per participant, to minimise bias resulting from non-independent treatment cycles, and to avoid statistical adjustments for repeated measures in women having two or more cycles (Dias *et al.*, 2008; Higgins *et al.*, 2022).

### NC-FET protocol

Women undergoing NC-FET attended on Day 10 of their menstrual cycle for a transvaginal ultrasound scan (Voluson^®^ S8, General Electrics, UK), and every two days thereafter. Once a dominant follicle measuring ≥14 mm was identified, participants began testing their urine twice daily to detect a spontaneous LH surge (Day 0). Where no LH surge was identified within 48 hr of visualisation of a dominant follicle, we repeated a transvaginal ultrasound to rule out ovulation and triggered ovulation as required with recombinant human chorionic gonadotropin (Ovitrelle^®^ 250 μg, Merck, UK) or urinary hCG (Gonasi^®^ 5000 international units, IBSA, UK) administered subcutaneously. Previously cryopreserved Day 5 or 6 blastocysts were thawed and transferred on Day 6 after a spontaneous LH surge, or Day 7 after hCG trigger injection. The decision to administer exogenous progesterone supplementation from the day of embryo transfer was left to the discretion of the attending clinicians and study participants. Where the local policy was to administer exogenous progesterone, this was given either as micronised progesterone vaginal capsules (Utrogestan^®^, Besins Healthcare, UK) or pessaries (Cyclogest^®^, L.D. Collins & Co., UK) 400 mg two times daily, from the day of embryo transfer. The decision on the progesterone regimen was made according to local protocols and/or clinician and patient preference. A urine pregnancy test was performed 16 days after embryo transfer, and pregnant women receiving progesterone supplementation continued it until 10–12 completed weeks of gestation. A transvaginal ultrasound scan was performed at ∼6–7 weeks of gestation to diagnose clinical pregnancy and confirm viability.

### HRT-FET protocol

In programmed FET cycles, the use of pituitary downregulation drugs was optional and varied according to local practice. Women who underwent downregulation received gonadotropin releasing hormone agonist injections (buserelin acetate, Suprecur^®^, Sanofi Aventis, UK) 0.5 mg once daily subcutaneously from Day 21 of the preceding menstrual cycle. We confirmed pituitary downregulation upon visualising quiescent ovaries and a thin endometrial lining (<4 mm) on transvaginal ultrasound.

Once downregulation was achieved, oestradiol valerate (Progynova^®^, Bayer, UK) was initiated at a dose of 2–4 mg three times daily, administered as oral tablets for at least 10 days. On Day 10 of oestradiol administration, we performed a transvaginal ultrasound scan to assess endometrial thickness (ET). In women whose ET was <9 mm, consideration was given to prolonging oral oestradiol treatment for another 4–7 days, or to adding a second route of administration, usually in the form of oestradiol transdermal patches (Evorel^®^, Theramex, UK) 100 μg once every 2–3 days. In participants undergoing HRT-FET without pituitary downregulation, the aforementioned regimen of oestradiol valerate was commenced on Day 2 of menstruation.

Following treatment with oestradiol valerate, all women undergoing HRT-FET cycles received progesterone supplementation for ∼120–124 hr prior to embryo transfer (i.e. 5 completed days of progesterone supplementation before FET). Progesterone was administrated either as micronised progesterone vaginal capsules (Utrogestan^®^, Besins Healthcare, UK) or pessaries (Cyclogest^®^, L.D. Collins & Co., UK) 400 mg two to three times daily, subcutaneous injection (Lubion^®^, Pharmasure, UK) 25 mg twice daily, or combined vaginal progesterone 400 mg twice daily and subcutaneous injection 25 mg once daily. Treatment outcomes were assessed in the same manner as that described for the NC-FET protocol.

### Embryo transfer

Previously cryopreserved Day 5 or 6 blastocysts were thawed on the day of embryo transfer and kept in a single-step medium (global^®^ total^®^, CooperSurgical, UK). Embryo transfer was performed under transabdominal ultrasound guidance (Voluson^®^ S8, General Electrics, UK) using a Sure-Pro^®^ or Sureview^®^ catheter (Wallace^®^, CooperSurgical).

### Progesterone measurement

All participating women underwent venepuncture on the day of FET, between 12:00 and 14:00 hours. In those receiving exogenous progesterone, the timing of blood sampling corresponded to ∼4–6 hr after the last dose of progesterone administration. We measured serum progesterone levels in a central laboratory using a high-throughput immunochemistry assay (Cobas^®^ e801, Roche Diagnostics, Germany). The measurement interval for serum progesterone ranged between 0.06 and 60.1 ng/ml. The intra- and inter-assay coefficients of variation ranged between 1.1% and 20.7% and 3.3% and 12.4%, respectively.

### Outcomes

Our primary outcome was the live birth rate per woman, defined as the birth of a live fetus at 24 or more weeks of gestation. The secondary outcomes were: the biochemical pregnancy rate per woman, defined as a positive urine or serum pregnancy test following embryo transfer; the clinical pregnancy rate per woman, defined as the presence of at least one gestational sac on transvaginal ultrasound; the implantation rate, defined as the number of gestational sacs seen on transvaginal ultrasound divided by the number of embryos transferred; the miscarriage rate per biochemical pregnancy, defined as any pregnancy lost before week 12 of gestation; and the ectopic pregnancy rate per biochemical pregnancy, defined as pregnancy outside the uterine cavity, diagnosed by ultrasound, surgical visualisation or histopathology (Zegers-Hochschild *et al.*, 2017).

### Follow-up

Participants were prospectively followed up until 31 December 2021, with final treatment outcome confirmed by telephone call.

### Sample size

Our systematic review showed that the ongoing pregnancy or live birth rate in women with inadequate progesterone levels was 34% versus 48% in participants with optimum progesterone levels (Melo *et al.*, 2021). To detect a 14% difference in the primary outcome of live birth with >99% power, we originally planned to recruit 900 participants. The study commenced shortly before the COVID-19 pandemic, however, and recruitment was thus substantially hindered by the impact of repeated clinic closures and the de-prioritisation of fertility services. We therefore opted to revise our sample size to a minimum of 396 women, allowing for a power of >70% and an alpha of 5%, and assuming a conservative 1:3 ratio of inadequate versus optimal progesterone levels based on existing literature (Yovich *et al.*, 2015; Labarta *et al.*, 2017, 2021a). We anticipated no loss to follow-up due to the mandatory requirement of reporting assisted reproductive treatment outcomes to the Human Fertilisation and Embryology Authority in the UK.

### Statistical analysis

All data analyses were performed using Stata Statistical Software (Release 17, TX, USA). We conducted unadjusted analyses for categorical variables using percentages and the chi-square test, and for continuous variables, we used the Student’s *t*-test. When comparing the same continuous variable (e.g. serum progesterone) across multiple groups (e.g. different progesterone administration routes), we used a one-way ANOVA test to identify any differences in the means between the various groups.

We conducted unadjusted analyses to investigate the association between serum progesterone deciles and FET outcomes among all participants. We also carried out multilevel mixed-effects multivariable logistic regression analyses to evaluate the association between serum progesterone levels and the treatment outcomes by adjusting for important confounders including participating centre, female age, BMI, parity, history of miscarriage, oocyte source (autologous versus donor), FET cycle type (NC-FET and HRT-FET), exogenous progesterone route, the use of PGT, the number of embryos transferred and embryo quality (top versus non-top quality). In this primary model, serum progesterone level was incorporated as a quadratic term to investigate a possible non-linear correlation with treatment outcomes. To further evaluate the effect of route of progesterone administration upon the association between serum progesterone and the probability of live birth, we conducted an adjusted subgroup analysis according to luteal phase support regimen (i.e. natural cycle with endogenous progesterone only and programmed cycle with vaginal progesterone only, subcutaneous progesterone only, or combined vaginal plus subcutaneous routes). In a second multivariable logistic regression model, serum progesterone was evaluated as a categorical variable, according to different deciles.

No imputation was required because there were no missing data for any of the confounders of interest. We expressed adjusted estimates as odds ratios (aOR) with 95% CI and judged results to incorporate high statistical confidence where the *P*-value was lower than 0.05.

## Results

### Study population and cycle characteristics

**Figure 1.**
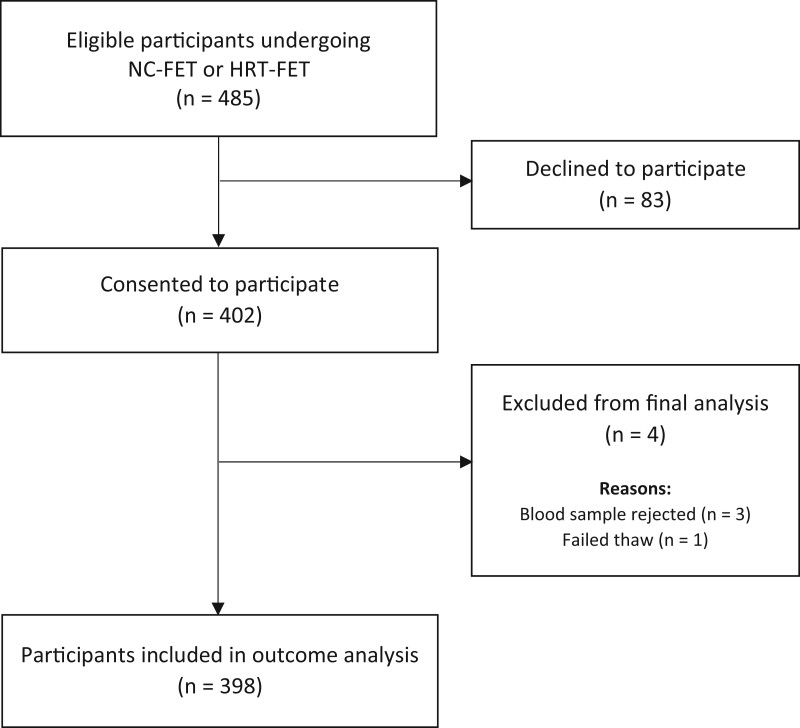
**Study flowchart.** FET, frozen embryo transfer; NC-FET, natural cycle frozen embryo transfer.

Between 2 January 2020 and 28 February 2021, of a total of 485 eligible women, 402 (82.9%) agreed to participate and were included in the study (Fig. 1). Table I contains details of the participants’ baseline and cycle characteristics, including a comparison between women with serum progesterone <10th centile (7.83 ng/ml) and all other participants. The mean participant age (± SD) was 35.3 ± 5.2 years, and the mean BMI ± SD was 25.2 ± 3.7 kg/m^2^. Most women underwent HRT-FET without pituitary downregulation (237/402, 59.0%) and received exogenous progesterone supplementation through the vaginal route (256/402, 63.7%). The blood samples of three women were deemed unsuitable for analysis by the laboratory, and we were thus unable to include them in the final outcome analysis. One further participant did not undergo embryo transfer due to a failed thaw. A total of 398 women were therefore included in the outcome analysis (Fig. 1). The mean level of serum progesterone on the day of FET was 14.9 ± 7.5 ng/ml. Women with serum progesterone <10th centile exhibited lower rates of nulliparity (53.9% versus 71.9%, *P *=* *0.025) and conventional IVF treatment (38.5% versus 51.2%, *P *<* *0.01) than participants whose serum progesterone was ≥10th centile.

**Table I:** Participant demographics and cycle characteristics.

Characteristic	Total cohort (n = 402)	Serum progesterone <10th centile (<7.83 ng/ml) (n = 39)	Serum progesterone ≥10th centile (≥7.83 ng/ml) (n = 363)	*P* value*
**Age (years),** mean ± SD	35.3 ± 5.2	33.7 ± 4.4	35.5 ± 5.2	0.053
**BMI (kg/m^2^),** mean ± SD	25.2 ± 3.7	25.4 ± 3.9	25.2 ± 3.7	0.750
**Ethnicity**				0.095
White	354 (88.1)	29 (74.4)	325 (89.5)	
South Asian	13 (3.2)	2 (5.1)	11 (3.0)	
Black	6 (1.5)	1 (2.6)	5 (1.5)	
Other	28 (7.0)	7 (17.9)	22 (6.0)	
**Cause of infertility**				0.085
Unexplained	117 (29.1)	7 (18.0)	110 (30.0)	
Male factor	86 (21.4)	8 (20.5)	78 (21.6)	
Female age/ovarian insufficiency	50 (12.4)	7 (18.0)	43 (11.9)	
Anovulation/PCOS	46 (11.4)	6 (15.4)	40 (11.1)	
Tubal pathology	28 (7.0)	5 (12.8)	23 (6.3)	
Other	75 (18.7)	6 (15.3)	69 (19.1)	
**Nulliparity**	282 (70.1)	21 (53.9)	261 (71.9)	0.025
**Previous miscarriage**	140 (34.8)	15 (38.5)	125 (34.4)	0.720
**Current smoker**	7 (1.7)	0	7 (1.9)	1.000
**Fresh cycle**				
IVF	201 (50.0)	15 (38.5)	186 (51.2)	<0.01
ICSI	195 (48.5)	21 (53.9)	174 (47.9)	
Historic cycle (unknown fertilisation method)	6 (1.5)	3 (7.6)	3 (0.9)	
**Donor oocyte use**	37 (9.2)	3 (7.7)	34 (9.4)	1.000
**PGT-A use**	30 (7.5)	1 (2.6)	29 (8.0)	0.340
**Time lapse technology use**	160 (39.8)	18 (46.2)	142 (39.1)	0.390
**Type of FET cycle**				0.590
NC-FET without exogenous progesterone	35 (8.7)	2 (5.1)	33 (9.1)	
NC-FET with exogenous progesterone^†^	10 (2.5)	0	10 (2.7)	
HRT-FET (no downregulation)	237 (59.0)	25 (64.1)	212 (58.4)	
HRT-FET (downregulation)	120 (29.8)	12 (30.8)	108 (29.8)	
**Progesterone route**				0.250
Vaginal only	256 (63.7)	32 (82.1)	224 (61.7)	
Injectable only	57 (14.2)	2 (5.1)	55 (15.1)	
Vaginal plus injectable	54 (13.4)	3 (7.7)	51 (14.1)	
No progesterone	35 (8.7)	2 (5.1)	33 (9.1)	
**Number of embryos transferred**				1.000
SET	363 (90.5)	36 (92.3)	327 (90.3)	
DET	38 (9.5)	3 (7.7)	35 (9.7)	
**Use of at least one top-quality embryo**	354 (88.3)	32 (82.1)	322 (89.0)	0.200
**Endometrial thickness before FET,** mean ± SD	9.6 ± 2.1	9.3 ± 1.6	9.6 ± 2.1	0.380
**Serum progesterone (ng/ml),** mean ± SD	14.9 ± 7.5	6.0 ± 1.7	15.8 ± 7.2	<0.001

All data are presented as frequency (%) unless stated otherwise.

* For comparison between serum progesterone <10th versus ≥10th centile.

† Vaginal progesterone capsules (n = 9) or pessaries (n = 1) at a dose of 400 mg twice daily.

DET, double embryo transfer; FET, frozen embryo transfer; NC-FET, natural cycle FET; PCOS, polycystic ovary syndrome; PGT-A, preimplantation genetic testing for aneuploidy; SET, single embryo transfer.

### Unadjusted analysis: all cycles

**Figure 2.**
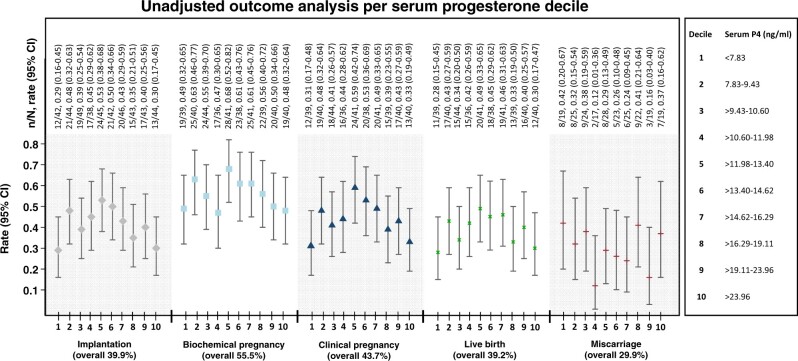
**Unadjusted analyses for the primary and secondary outcomes according to serum progesterone deciles.** n, number of events; N, total of women analysed within the corresponding serum progesterone decile; P_4_, progesterone.

Overall, the live birth rate was 39.2% (156/398), the clinical pregnancy rate was 43.7% (174/398), the implantation rate was 39.9% (174/436), the biochemical pregnancy rate was 55.5% (221/398) and the miscarriage rate was 29.9% (66/221). Figure 2 shows the unadjusted analyses of FET outcomes by serum progesterone decile. The live birth, clinical pregnancy and implantation rates were highest in women whose serum progesterone ranged between the 40th and 50th centiles (>11.98–13.4 ng/ml; live birth rate 48.8% [20/41], clinical pregnancy rate 58.5% [24/41] and implantation rate 53.3% [24/45]). The miscarriage rate was lowest in participants with serum progesterone levels ranging between the 30th and 40th centiles (>10.6–11.98 ng/ml; miscarriage rate 11.8% [2/17]). There were no ectopic pregnancies identified in the study population.

### Adjusted analysis: all cycles

**Figure 3.**
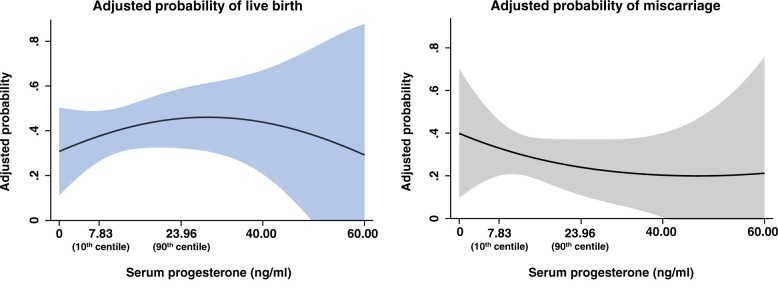
**Multivariable regression model describing the adjusted probability of live birth and miscarriage according to serum progesterone levels.** The dark line represents the mean adjusted probability, with respective 95% CI in shading. Model adjusted for recruiting centre, age, BMI, oocyte source, use of preimplantation genetic testing, history of miscarriage, parity, type of cycle (natural versus programmed), progesterone route of administration, number of embryos transferred and embryo quality (top versus non-top).

**Figure 4.**
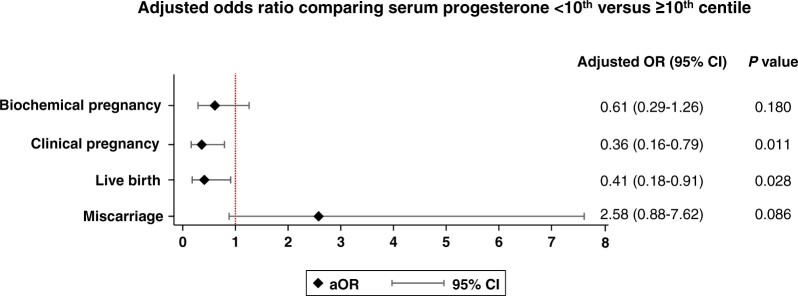
**Forest plots describing the adjusted effect of progesterone levels <10th centile (<7.83 ng/ml) on the main clinical outcomes of clinical pregnancy, live birth and miscarriage rates in comparison with serum progesterone ≥10th centile (≥7.83 ng/ml, reference).** Multivariable logistic regression model adjusted for recruiting centre, age, BMI, oocyte source, use of preimplantation genetic testing, history of miscarriage, parity, type of cycle (natural versus programmed), route of progesterone administration, number of embryos transferred and embryo quality (top versus non-top). aOR, adjusted odds ratio.

When serum progesterone was analysed as a continuous covariate in the multivariable logistic regression model including all routes of progesterone administration, the mean probability of live birth increased non-linearly from 37.6% (95% CI 26.3–48.9%) to 45.5% (95% CI 32.1–58.8%) as serum progesterone rose between the 10th and 90th centiles (Fig. 3). Within the same range of serum progesterone levels, the mean probability of miscarriage decreased non-linearly from 33.0% (95% CI 20.0–46.0%) to 24.0% (95% CI 10.9–37.1%). The wide CIs precluded the identification of an optimum range of progesterone levels associated with treatment success. However, serum progesterone levels lower than 7.83 ng/ml (10th centile) were confidently associated with a 59% reduction in live birth rate (aOR 0.41, 95% CI 0.18–0.91, *P *=* *0.028), a 64% lower chance of clinical pregnancy (aOR 0.36, 95% CI 0.16–0.79, *P *=* *0.011) and a tendency towards higher odds of miscarriage (aOR 2.58, 95% CI 0.88–7.62, *P *=* *0.086) (Fig. 4).

### Unadjusted analysis according to progesterone regimen

The mean serum progesterone level was highest in women using the injectable route (21.10 ± 8.64 ng/ml) and lowest in those receiving vaginal progesterone supplementation in HRT-FET cycles (12.69 ± 5.84 ng/ml) (*P *<* *0.001) (Table II). We did not identify a difference in clinical outcomes for the comparisons of NC-FET with exogenous progesterone versus no progesterone, injectable versus no progesterone, and combined routes (vaginal and injectable) versus no progesterone. However, in comparison to NC-FET without exogenous progesterone, HRT-FET cycles using vaginal progesterone supplementation were associated with higher implantation rates (OR 2.29, 95% CI 1.01–5.54, *P *=* *0.03), increased odds of biochemical (OR 2.33, 95% CI 1.06–5.19, *P *=* *0.02) and clinical pregnancy (OR 2.60, 95% CI 1.15–6.33, *P *=* *0.01), and a tendency towards increased odds of live birth (OR 2.04, 95% CI 0.90–4.95, *P *=* *0.07).

**Table II:** Treatment outcomes according to progesterone regimen.

	NC-FET no progesterone (n = 35)	Crude OR (95% CI)	NC-FET vaginal P_4_ (n = 10)	Crude OR (95% CI)^*^	HRT-FET vaginal P_4_ (n = 245)	Crude OR (95% CI)	HRT-FET subcutaneous P_4_ (n = 57)	Crude OR (95% CI)	HRT-FET combined vaginal and subcutaneous P_4_ (n = 54)	Crude OR (95% CI)	*P* value for ANOVA
**Serum progesterone (ng/ml),** mean ± SD	14.75 ± 4.74	–	14.46 ± 3.40	–	12.69 ± 5.84	–	21.10 ± 8.64	–	18.14 ± 9.75	–	<0.001
**Implantation rate**	10/35 (28.6)	Ref	0/10 (0)	0 (0–1.05), *P *=* *0.06	130/272 (47.8)	2.29 (1.01–5.54), *P = *0.03	17/60 (28.3)	0.99 (0.40–2.45), *P *=* *1.00	23/62 (37.1)	1.47 (0.61–3.56), *P *=* *0.50	–
**Biochemical pregnancy**	14 (40.0)	Ref	2 (20.0)	0.38 (0.04–2.34), *P *=* *0.24	149 (60.8)	2.33 (1.06–5.19), *P *=* *0.02	27 (47.4)	1.35 (0.58–3.17), *P *=* *0.52	31 (57.4)	2.02 (0.85–4.80), *P *=* *0.13	–
**Clinical pregnancy**	10 (28.6)	Ref	0 (0)	0 (0–1.05), *P *=* *0.06	125 (51.0)	2.60 (1.15–6.33), *P *=* *0.01	17 (29.8)	1.06 (0.42–2.69), *P *=* *1.00	23 (42.6)	1.86 (0.75–4.61), *P *=* *0.261	–
**Miscarriage**	4 (28.6)	Ref	2 (100)	Inestimable	38 (25.5)	0.86 (0.23–3.96), *P *=* *0.80	11 (40.7)	1.72 (0.43–6.90), *P *=* *0.51	11 (35.5)	1.38 (0.35–5.43), *P *=* *0.74	–
**Live birth**	10 (28.6)	Ref	0 (0)	0 (0–1.05), *P *=* *0.06	110 (43.1)	2.04 (0.90–4.95), *P *=* *0.07	16 (28.1)	0.98 (0.38–2.48), *P *=* *1.00	20 (37.0)	1.47 (0.59–3.68), *P *=* *0.49	–

All data are presented as frequency (%) unless otherwise stated.

* Odds ratios were calculated with the Cornfield approximation where event numbers were 0.

FET, frozen embryo transfer; NC, natural cycle; OR, odds radio; P_4_, progesterone; Ref, reference group.

### Adjusted analysis according to progesterone regimen

**Figure 5.**
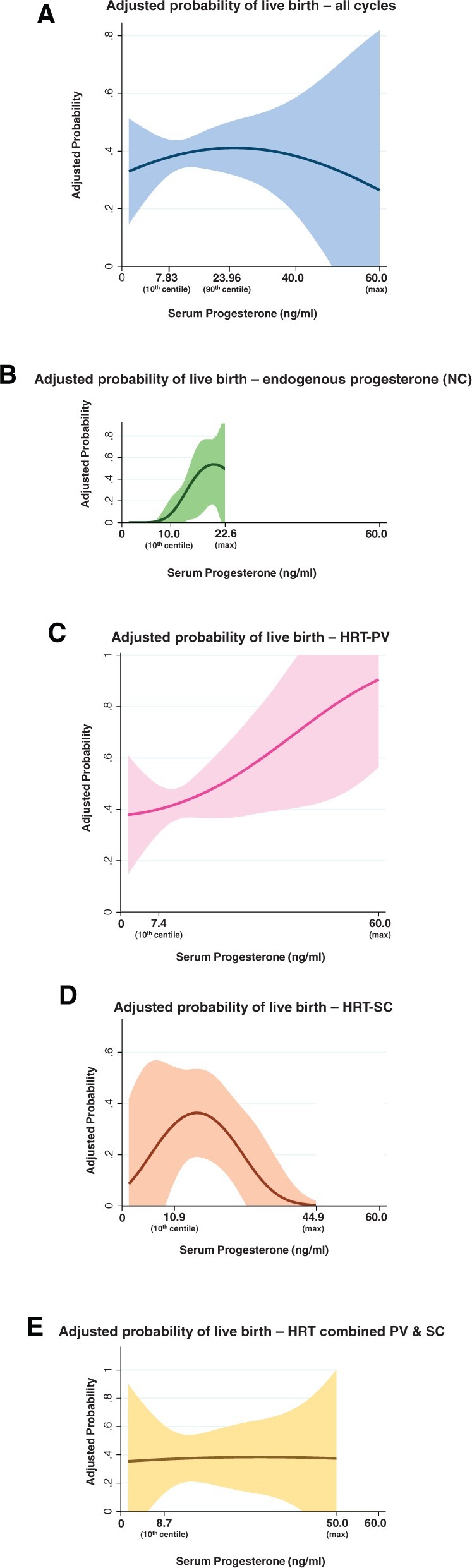
**Multivariable regression model describing the association between serum progesterone levels and the adjusted probability of live birth according to route of progesterone administration.** (**A**) All cycles, irrespective of route. (**B**) Natural cycle FET. (**C**) HRT cycle, vaginal route. (**D**) HRT cycle, subcutaneous route. (**E**) HRT cycle, combined vaginal and subcutaneous routes. The dark line represents the mean adjusted probability, with respective 95% CI in shading. (A) Model adjusted for recruiting centre, age, BMI, oocyte source, use of preimplantation genetic testing, history of miscarriage, parity, type of cycle (natural versus programmed), progesterone route of administration and number of embryos transferred and embryo quality (top versus non-top). (B–E) Model adjusted for recruiting centre, age, BMI, oocyte source, use of preimplantation genetic testing, history of miscarriage, parity, number of embryos transferred and embryo quality (top versus non-top). FET, frozen embryo transfer; NC, natural cycle; PV, vaginal route; SC, subcutaneous route.

The association between serum progesterone levels and the probability of live birth appeared to vary according to the FET cycle regimen (Fig. 5). In NC-FET cycles relying exclusively on endogenous progesterone synthesis, the mean probability of live birth increased to a maximum of 53.9% (95% CI 23.7–84.1%) as progesterone levels reached 20.3 ng/ml, and declined slightly beyond this level of serum progesterone. In women undergoing HRT cycles with vaginal progesterone, the mean probability of live birth increased approximately linearly as serum progesterone rose. Women having HRT-FET with only subcutaneous exogenous progesterone exhibited an increase in the mean adjusted probability of live birth up to a level of 16.3 ng/ml (maximum probability 36.4%, 95% CI 19.2–53.5%), beyond which treatment success appeared to decline steeply. Lastly, the combined use of vaginal and subcutaneous progesterone resulted in little evidence of variation in the mean probability of live birth according to serum progesterone, ranging between 36% and 38.4%. However, CIs were wide for all progesterone routes. The low number of women having NC-FET with exogenous progesterone (n = 10) precluded multivariable logistic regression investigating the association between serum progesterone levels and the probability of live birth in this subgroup.

## Discussion

### Summary of evidence

In this prospective multicentre cohort study, we investigated the association between serum luteal progesterone and treatment outcomes in women undergoing FET across eight fertility clinics in the UK. Our results support the hypothesis that low serum progesterone is associated with reduced treatment success in FET. Below the 10th centile (∼7.8 ng/ml), the rates of clinical pregnancy and live birth were reduced, and the risk of miscarriage tended to increase. In women having a natural cycle without exogenous progesterone or HRT-FET treatment using vaginal progesterone, our adjusted analyses suggested a positive correlation between serum progesterone levels and the mean probability of live birth. However, this association appeared to be non-linear for women receiving subcutaneous progesterone only, in whom both lower and higher serum progesterone levels were associated with a decrease in the mean probability of live birth. Further, the mean probability of live birth remained stable in women receiving combined vaginal and subcutaneous progesterone, irrespective of serum progesterone levels.

The hypothesis that FET success may be affected by an insufficient luteal phase has only recently been evaluated. In a systematic review of studies investigating the association between serum luteal progesterone and FET outcomes, we found that for cut-off levels below 10 ng/ml, women with lower serum progesterone experienced fewer ongoing pregnancies or live births, a reduction in clinical pregnancy rate and increased risk of miscarriage (Melo *et al.*, 2021). These findings were limited to studies where only vaginal progesterone had been used in HRT-FET cycles, however, and there was a paucity of prospective evidence on NC-FET and HRT-FET treatment using non-vaginal routes of progesterone administration. In addition, our review identified a need for prospective data investigating serum progesterone in a continuous fashion rather than focusing on the ‘high’ versus ‘low’ dichotomy. The present study addresses the existing knowledge gaps by including all women undergoing FET treatment, regardless of cycle type or progesterone administration route, and by approaching serum progesterone as a continuous variable when adjusting for confounders. Our results support the hypothesis that there is a minimum level of serum progesterone required for successful implantation and placentation in FET cycles, in addition to offering further insight into the effect-modifying role exerted by different routes of progesterone administration.

Although progesterone measurements below the 10th centile were associated with worse outcomes in this cohort, our adjusted percentile analyses did not categorically demonstrate a linear increase in live birth rates as serum progesterone measurements rose beyond this level. The adjusted analyses suggest that serum progesterone levels above the 90th centile may have in fact been associated with a reduction in the mean probability of live birth and a slight increase in the risk of miscarriage, although the wide CIs precluded any conclusive determinations of effect size. Whether high serum progesterone may be detrimental to pregnancy outcomes has been the subject of some debate, and the evidence in FET cycles remains uncertain (Kofinas *et al.*, 2015; Yovich *et al.*, 2015; Alsbjerg *et al.*, 2020; Melo *et al.*, 2021). In a study by Liang *et al.* (2018), excessive progesterone in pregnant mice was found to be just as deleterious to endometrial receptivity and decidualisation as low progesterone. The authors did not evaluate serum progesterone levels, however, and focused instead on the intravenous administration of successively higher progesterone doses.

There has been substantial controversy in the literature on the optimum route of progesterone administration in FET. In a seminal three-arm trial by Devine *et al.* (2021) including 1125 women undergoing FET, the authors randomly allocated participants to endometrial preparation using daily intramuscular progesterone (50 mg), daily vaginal micronised progesterone plus intramuscular progesterone every 3 days, or daily vaginal micronised progesterone only. Women in the vaginal-only arm had a lower live birth rate than those in the other two groups, driven by an increased incidence of miscarriage identified in an interim analysis (Devine *et al.*, 2018). This resulted in the premature termination of the vaginal-only treatment arm. However, the relatively low dose of vaginal progesterone (200 mg twice daily) may have contributed to these results, in which case the use of a systemic route of administration may have led to luteal rescue in the combination therapy arm. Crucially, the incidence of miscarriage per pregnancy was highest (80%), and the live birth rate per participant was lowest (33%) in women with very low serum progesterone (<1 ng/ml) measured 2 weeks after FET, although the investigators did not present analyses adjusted for the route of administration. In our study, women who exclusively received injectable progesterone (25 mg subcutaneously twice daily) had the highest circulating progesterone levels. Yet in the unadjusted analysis according to progesterone administration route, those who only took injectable progesterone also experienced the lowest number of live births and clinical pregnancies. In addition, the adjusted analyses according to route of administration suggest that the mean probability of live birth increased as serum progesterone levels rose to 16.3 ng/ml, beyond which the mean probability of treatment success declined. This appears to be in stark contrast with the findings of the aforementioned randomised controlled trial, and it may be explained in part by the difference in pharmacokinetics between the intramuscular and subcutaneous routes.

The use of subcutaneous progesterone for luteal phase support has become increasingly common in the past decade, although the vaginal and intramuscular routes remain the most used worldwide (Shoham *et al.*, 2021). While data comparing different dosages of the aqueous subcutaneous (25 mg OD versus 50 mg OD) have not identified differences in endometrial decidualisation (de Ziegler *et al.*, 2013), there remains a paucity of studies investigating pregnancy outcomes in women having luteal phase support with aqueous versus oil-based formulations. Evidence suggests that the subcutaneous administration of 25 mg progesterone in aqueous solution results in maximum plasma concentrations within 2 hr, rapidly followed by a drop to <10 ng/ml in some women within the first 8 hr of administration, whereas the intramuscular route in oil solution confidently leads to sustained serum levels >20–30 ng/ml for at least 24 hr (Sator *et al.*, 2013). In our cohort, it is possible that by performing venepuncture 4–6 hr after the last subcutaneous injection, we may have obtained high serum progesterone measurements that failed to reflect potentially subtherapeutic levels shortly thereafter, extending to low trough concentrations right before the next progesterone dose. However, this does not adequately explain why live birth rates decreased substantially beyond serum progesterone levels of 16.3 ng/ml in this subgroup of women. It is possible that such a phenomenon may have stemmed from decreased responsiveness of uterine receptors to increasing systemic progesterone levels, especially in the absence of a compensatory effect otherwise exerted by vaginal progesterone and its ‘first-uterine pass’ effect (Chrousos *et al.*, 1986). In addition, recent data suggest that there may not be an association between serum progesterone levels, uterine progesterone levels and endometrial receptivity as measured by transcriptomic analysis (Labarta *et al.*, 2021b).

In women undergoing HRT-FET with vaginal progesterone, recent studies have suggested that additional supplementation using a second route of administration may lead to luteal rescue where serum progesterone levels are low (Álvarez *et al.*, 2021; Yarali *et al.*, 2021; Labarta *et al.*, 2021a). Álvarez *et al.* (2021) conducted a prospective non-randomised interventional study where 453 women having programmed FET with vaginal progesterone underwent venepuncture and serum measurement of progesterone levels on the day before euploid embryo transfer. Those whose serum progesterone was <10.6 ng/ml received additional subcutaneous progesterone from the day of FET and experienced similar rates of clinical pregnancy, live birth and miscarriage to those of participants with progesterone levels ≥10.6 ng/ml. In a subsequent study, Labarta *et al.* (2022) retrospectively analysed 2275 women having HRT-FET with micronised vaginal progesterone. The authors added subcutaneous progesterone for luteal phase support in women whose serum progesterone was <9.2 ng/ml on the day of FET, and demonstrated non-inferior clinical outcomes in comparison to the group where serum progesterone was ≥9.2 ng/ml. In both studies, the intervention was based upon a single venepuncture and serum progesterone measurement, suggesting that once an additional route of progesterone administration has been commenced, there may be little value in obtaining serial progesterone levels. The results of our study are in line with these findings by demonstrating that in women who received combined vaginal and subcutaneous progesterone, the probability of live birth exhibited little variation in relation to serum progesterone levels. Although some may argue that these results support the universal use of combined vaginal and subcutaneous progesterone in HRT-FET, it must be noted that such an approach might also result in added adverse events and treatment costs.

The mechanisms through which different routes of exogenous progesterone may act synergistically have not been fully clarified. In natural conception, the corpus luteum secretes progesterone into microvessels that feed into the pelvic vasculature and, in turn, the systemic circulation (Takasaki *et al.*, 2009; Han *et al.*, 2019). It is thus plausible to consider that low serum progesterone levels may constitute a proxy marker of a hypoprogestogenic state and insufficient luteal support (Verhaegen *et al.*, 2012). In the uterus, progesterone has been shown to facilitate decidualisation and embryo implantation by inducing an optimum immune-endocrine milieu within the endometrial lining (Shah *et al.*, 2019; Pereira *et al.*, 2021). Progesterone also acts upon the myometrium, reducing its contractility and therefore leading to a state of mechanical quiescence which is additionally thought to aid early placentation (Mueller *et al.*, 2006; Moliner *et al.*, 2021; Pereira *et al.*, 2021). Systemically, progesterone exerts a regulatory role upon the innate and acquired immune responses, resulting in an inhibition of cytotoxicity and the induction of immune tolerance required for successful implantation (Szekeres-Bartho, 2018; Shah *et al.*, 2019). The bioavailability of progesterone administered vaginally varies according to many factors including dose, frequency of administration, the woman’s age and BMI, the saturability of vaginal and uterine progesterone receptors, and the ability of the epithelial lining to absorb the drug into the systemic circulation (MacLaughlin and Richardson, 1976; Levy *et al.*, 1980; Archer *et al.*, 1995; González-Foruria *et al.*, 2020; Whynott *et al.*, 2021). There is, hence, biological plausibility to the hypothesis that in women with a low level of circulating progesterone, the addition of a systemic route of exogenous progesterone administration may compensate, at least partly, for low absorption rates where there is impaired local absorption. By the same token, it is also reasonable to postulate that injectable progesterone in isolation, while capable of achieving high serum progesterone levels, may not guarantee sufficient uterine supply, possibly exerting a deleterious effect on treatment success.

Recent evidence suggests that HRT-FET cycles are associated with a substantial increase in the risk of hypertensive disorders of pregnancy compared to NC-FET, possibly owing to the lack of a corpus luteum (von Versen-Höynck *et al.*, 2019; Wang *et al.*, 2020). It is thought that luteal secretory products other than progesterone, including relaxin-2, may exert a modulatory effect upon the uterine vasculature and thus facilitate normal implantation (Pereira *et al.*, 2021). These data have led some researchers to support a move towards favouring the use of NC-FET for improved safety and non-inferior effectiveness compared to HRT-FET (von Versen-Höynck and Griesinger, 2022). While studies investigating the association between serum progesterone and NC-FET outcomes remain scarce, observational data show that low serum progesterone may be nefarious to live birth rates in NC-FET (Gaggiotti-Marre *et al.*, 2020). These findings add strength to the concept of luteal phase defect in NC-FET and are further corroborated by evidence suggesting that LPS in NC-FET results in improved treatment outcomes (Mizrachi *et al.*, 2021).

### Strengths and limitations

The prospective nature of this study, its multicentre design and the blinding of participants and personnel are its major strengths. We collected extensive demographic and endpoint data for all participants, allowing for robust adjusted analyses that confidently identified an independent effect of serum progesterone upon FET outcomes accounting for possible confounders such as the inclusion of different FET regimens. Our findings corroborate those of other studies where only vaginal progesterone was administered (Labarta *et al.*, 2017, 2022), in addition to offering further insight into the association between serum progesterone and live birth in NC-FET and HRT-FET cycles using non-vaginal routes of progesterone administration.

The principal limitation of our study is that instead of the originally planned 900 participants, we could only recruit 402 women due to constraints linked to the COVID-19 pandemic. We based our revised calculation upon a systematic review of the literature, however, and were able to reach satisfactory power (>70%) to detect a meaningful difference between optimal and inadequate serum progesterone levels. In addition, our cohort did not include women receiving oral (e.g. dydrogesterone) and rectal progestogens, and therefore our findings may not be generalisable to these two routes of administration. Although dydrogesterone has been increasingly administered in recent years for luteal phase support, this remains uncommon in the UK. Importantly, Vuong *et al.* (2021) prospectively compared the use of micronised vaginal progesterone alone versus in combination with oral dydrogesterone and found higher live birth rates in women receiving combined therapy. This requires further corroboration, because the oral route may offer improved convenience to women undergoing treatment. Nonetheless, allocation to the two treatment arms was not based upon serum progesterone measurements, and there was in fact no difference in serum progesterone levels between the two groups (Vuong *et al.*, 2021). The use of rectal progesterone has been shown to result in similar serum levels to the vaginal route (Nillius and Johansson, 1971). Although there is evidence on the effect of the combined rectal plus vaginal routes of progesterone upon FET outcomes (Alsbjerg *et al.*, 2020), the former has not been studied in isolation.

An additional limitation of our study relates to its pragmatic nature. We pre-specified to include all women undergoing FET, regardless of treatment protocol. Prospectively collected data allowed for robust multivariable logistic regression analyses, yet it is possible that some residual confounding may have remained. For example, the choice of FET regimen was left to the discretion of the attending physician and participants, which might have been influenced by participants’ characteristics (e.g. BMI or history of pregnancy loss). However, our adjusted analyses considered such confounders, thus minimising the risk of bias. Furthermore, among women receiving exclusively vaginal progesterone (n = 256), a minority (n = 12) had the 400 mg TDS regimen and 244 women received the 400 mg BD regimen. We conducted a post hoc sensitivity analysis for women receiving vaginal progesterone adjusting for dose, and this did not change the primary findings.

Lastly, the timing of serum progesterone measurement varied between different participating centres, and it was not possible to account for this due to the pragmatic nature of our study. In women who received exogenous progesterone, this may have not affected the reliability of serum progesterone results, because most progesterone formulations lead to a steady-state concentration of progesterone within 24–72 hr (Levy *et al.*, 2000; Corleta *et al.*, 2004; Paulson *et al.*, 2014; Cometti, 2015). In participants undergoing NC-FET, the accuracy of serum progesterone measurements may have been affected by the pulsatile fashion in which the corpus luteum secretes progesterone (Filicori *et al.*, 1984), although the study by Gaggiotti-Marre *et al.* (2020) suggested a good correlation between serum progesterone and treatment outcomes in NC-FET.

## Conclusions

Our results corroborate existing evidence suggesting that low serum progesterone levels hinder FET success. However, the relationship between serum progesterone and the probability of live birth appears to be non-linear in women receiving exclusively subcutaneous progesterone, suggesting that in this subgroup of women, high serum progesterone may also be detrimental to treatment success.

## Data Availability

The data underlying this article will be shared on reasonable request to the corresponding author.
